# Supplementary material for: Culm Morphological Analysis in Moso Bamboo Reveals the Negative Regulation of Internode Diameter and Thickness by Monthly Precipitation
Source: Plants (Basel). 2024 May 28;13(11):1484. doi: 10.3390/plants13111484 (PMC11175016; doi:10.3390/plants13111484)
Supplement: Supplementary file 1 [file plants-13-01484-s001.zip › plants-2941524-supplementary.pdf]

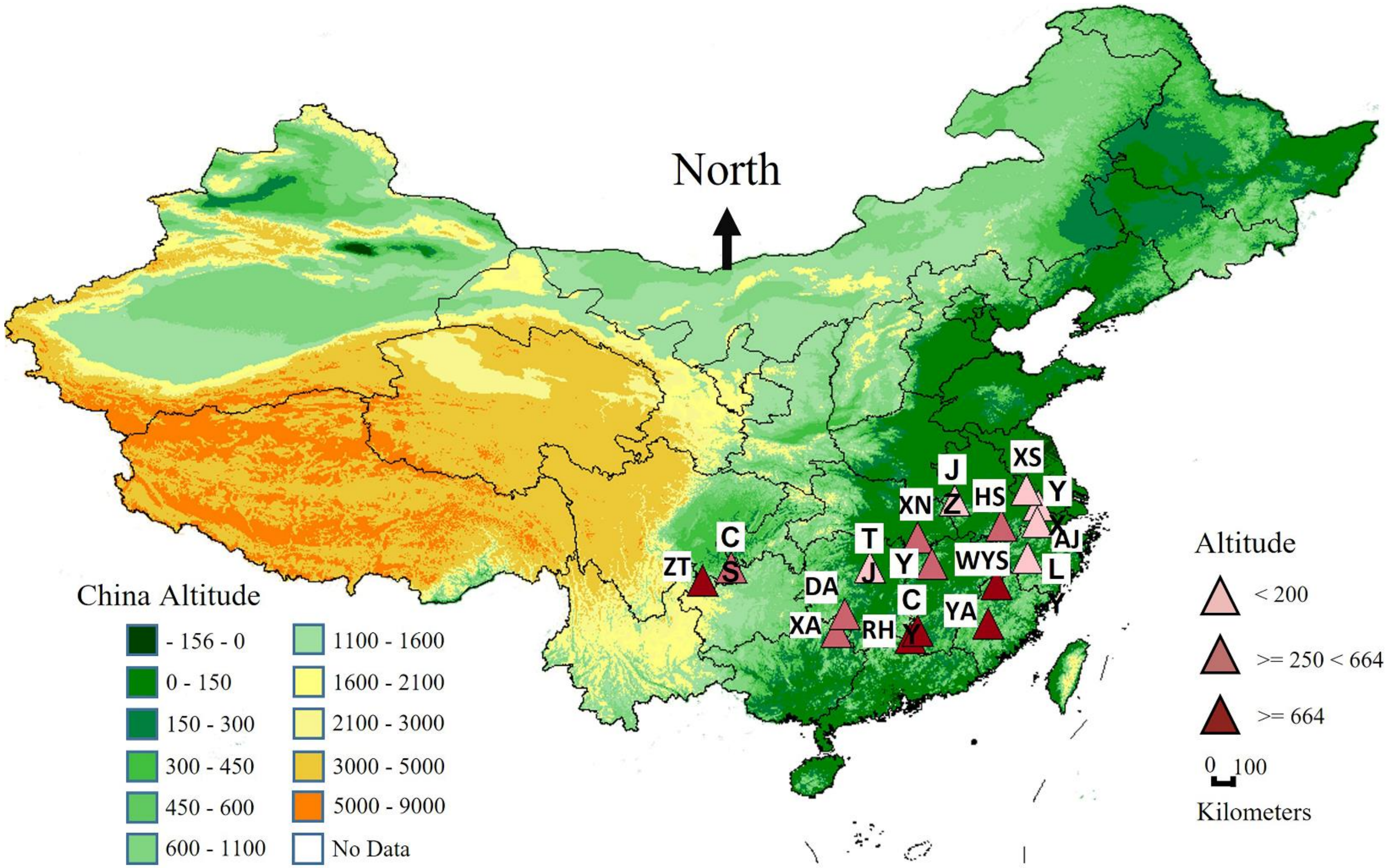

Figure S1. A geographical map illustrating the distribution of the 16 Moso bamboo populations across 11 provinces of China. Each abbreviation code, such as AJ, CS, CY, DA, HS, JZ, LY, RH, TJ, WYS, XA, XN, YA, YF, YX, and ZT, corresponds to one of the 16 sampling regions in China. For details about the abbreviation codes, please refer to Table S1. The map was generated using DIVA-GIS, a free computer program for geographic information (https://www.diva-gis.org/gdata).

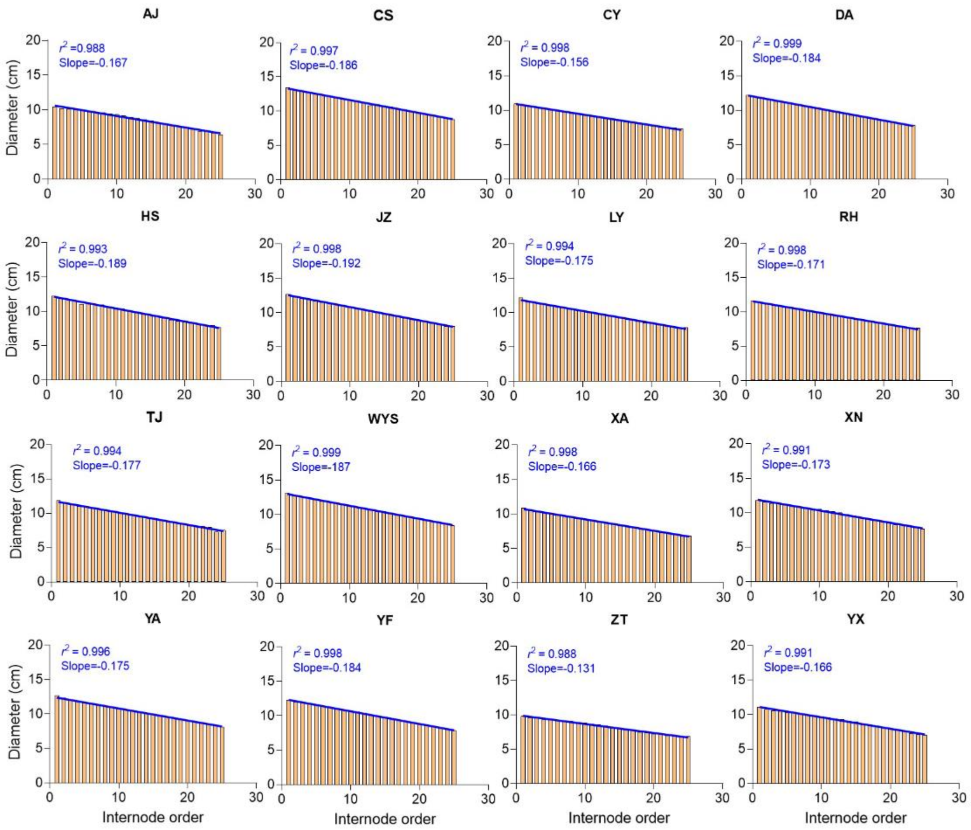

Figure S2. The correlation analysis illustrates the relationship strength (r^2) and slope values between the internode diameter and internode order across various Moso bamboo populations. Each abbreviation code, such as AJ, CS, CY, etc., represents 16 sampling regions in China. The r^2 value represents the coefficient of determination, indicating the strength of the correlation, while the negative slope value indicates the rate of change in the dependent variable (internode diameter) for a one-unit increase in the independent variable (internode order). For example, the slope value of -0.131 indicates that for every one-unit increase in the internode order, the internode diameter decreases by 0.131 units on average, as observed in the ZT population.

Table S1. List of sixteen sampling regions of Moso bamboo in China with geographical coordinates, average annual precipitation, and annual mean temperature.

S.No.	Name of the sampling regions (Abbreviation)	Geographical coordinates (Longitude and latitude)	Altitude (m)	Average annual precipitation (mm)	Annual mean temperature (°C)
1	Yinkeng Village, Anji, Zhejiang province (AJ)	119°37′57″E, 30°30′40″N	161	1423	17.0
2	Longgong Village, Yong’an, Fujian province (YA)	117°27′23″E, 25°58′20″N	791	1815	19.3
3	Qianshan National Forest Park, Xianning, Hubei province (XN)	114°18′57″E, 29°48′56″N	250	1588	17.2
4	Huangshan Public Forest Farm, Huangshan, Anhui province (HS)	118°19′19″E, 30°07′33″N	491	1569	15.3
5	Shunhuang Mountain National Forest Park, Dong'an, Hunan province (DA)	111°02′24″E, 26°24′13″N	452	1600	16.8
6	Chengdun Village, Wuyishan, Fujian province (WYS)	117°50′22″E, 27°42′40″N	754	1927	18.3
7	Dagang Experimental Forest Farm of Jiangxi Agricultural University, Yifeng, Jiangxi province (YF)	114°56′12″E, 28°37′27″N	415	1767	16.7
8	Lingxi village, Renhua, Guangdong province (RH)	114°0′38″E, 25°18′23″N	664	1640	19.6
9	Xikou Village, Longyou, Zhejiang province (LY)	119°12′23″E, 28°53′23″N	249	1603	17.1
10	Yangling National Forest Park, Chongyi, Jiangxi province (CY)	114°18′39″E, 25°38′38″N	752	1615	17.8
11	Yaozhu Town, Xing'an, Guangxi province (XA)	110°25′41″E, 25°52′23″N	460	1893	18.0

12	Yaotang Village, Jinzhai, Anhui province (JZ)	115°58′08″E, 31°26′42″N	216	1420	15.9
13	Nanyue Mountain Villa, Yixing, Jiangsu province (YX)	119°43′42″E, 31°15′08″N	34	1294	15.7
14	Zhuhai National Forest Park, Chishui, Guizhou province (CS)	105°59′41.433″E, 28°28′15.266″N	567	1196	15.6
15	Huangjingtang Bamboo Forest, Taojiang, Hunan province (TJ)	112°11′18″E, 28°28′39″N	247	1400	17.1
16	Hebatou Haiziping National Forest Park, Zhaotong, Yunnan Province (ZT)	104°42′52″E, 27°53′17″N	1100	843	15.0

Table S2. Statistical measures of culm heights across various Moso bamboo populations. Each column represents a distinct Moso bamboo population, denoted by codes like AJ, CS, CY, etc., representing 16 sampling regions in China.

	**AJ**	**CS**	**CY**	**DA**	**HS**	**JZ**	**LY**	**RH**	**TJ**	**WYS**	**XA**	**XN**	**YA**	**YF**	**ZT**	**YX**
**Number of values**	30	27	30	29	30	30	30	30	30	30	30	30	30	30	30	30
**Minimum (m)**	8.00	16.0	11.7	12.9	12.2	12.8	11.2	11.2	11.2	11.8	11.0	12.5	13.6	11.6	11.2	12.0
**Maximum (m)**	10.9	19.4	14.8	16.9	17.4	18.1	15.7	18.0	15.9	22.1	15.0	17.3	18.0	16.6	16.3	16.8
**Range (m)**	2.90	3.40	3.12	4.00	5.20	5.32	4.50	6.80	4.70	10.3	4.00	4.80	4.40	5.00	5.10	4.76
**Mean (m)**	9.67	17.5	13.3	15.1	14.8	15.2	13.0	15.0	13.7	16.1	12.9	15.3	16.1	14.6	14.0	13.8
**Std. Deviation**	0.55	0.99	0.80	1.07	1.29	1.36	1.14	1.39	1.22	1.81	1.04	1.20	1.05	1.07	1.26	1.11
**Coefficient of variation (%)**	5.73	5.70	6.07	7.12	8.68	8.97	8.74	9.26	8.91	11.20	8.06	7.83	6.50	7.32	9.04	8.03

Table S3. Statistical measures of culm heights under the first branch across various Moso bamboo populations. Each column represents a distinct Moso bamboo population, denoted by codes like AJ, CS, CY, etc., representing 16 sampling regions in China.

	AJ	CS	CY	DA	HS	JZ	LY	RH	TJ	WYS	XA	XN	YA	YF	ZT	YX
**Number of values**	30	30	30	31	30	30	30	30	30	30	30	30	30	30	30	31
**Minimum (m)**	3.67	5.25	4.10	5.10	4.10	4.00	3.11	4.23	3.70	5.22	3.84	4.50	2.35	3.60	3.55	4.00
**Maximum (m)**	6.16	8.90	7.74	8.18	8.50	8.70	6.96	9.47	9.30	9.33	6.76	8.40	10.5	8.80	7.50	8.31
**Range (m)**	2.49	3.65	3.64	3.08	4.40	4.70	3.85	5.24	5.60	4.11	2.92	3.90	8.15	5.20	3.95	4.31
**Mean (m)**	4.91	7.33	5.19	6.39	6.53	5.99	5.12	6.65	6.11	7.02	5.31	6.78	7.67	6.41	5.82	5.44
**Std. Deviation**	0.59	0.86	0.97	0.73	0.87	1.26	0.98	1.37	1.24	1.07	0.69	1.10	1.46	1.08	0.96	0.87
**Coefficient of variation (%)**	11.9	11.7	18.8	11.5	13.3	21.0	19.2	20.6	20.3	15.3	13.1	16.2	19.1	16.8	16.5	15.9

Table S4. The ratio of culm heights under the first branch to total culm height across various Moso bamboo populations. Each column represents a distinct Moso bamboo population, denoted by codes like AJ, CS, CY, etc., representing 16 sampling regions in China.

	AJ	CS	CY	DA	HS	JZ	LY	RH	TJ	WYS	XA	XN	YA	YF	ZT	YX
**Number of values**	30	30	30	31	30	30	30	30	30	30	30	30	30	30	30	31
**Minimum (%)**	0.38	0.32	0.32	0.35	0.30	0.28	0.27	0.33	0.33	0.29	0.31	0.32	0.14	0.27	0.24	0.32
**Maximum (%)**	0.62	0.63	0.53	0.51	0.60	0.50	0.48	3.05	0.68	0.56	0.50	0.55	0.62	0.55	0.56	0.67
**Range**	0.24	0.31	0.21	0.16	0.30	0.22	0.21	2.72	0.35	0.27	0.19	0.24	0.48	0.29	0.32	0.36
**Mean (%)**	0.51	0.43	0.39	0.42	0.44	0.39	0.39	0.53	0.45	0.44	0.41	0.44	0.48	0.44	0.42	0.40
**Std. Deviation**	0.06	0.05	0.05	0.03	0.05	0.06	0.06	0.48	0.08	0.06	0.04	0.05	0.09	0.05	0.08	0.06
**Coefficient of variation (%)**	11.9	12.2	18.8	11.5	13.3	21.0	19.2	76.4	20.3	15.3	13.1	16.2	19.3	16.8	16.5	16.6

Table S5. The total number of internodes under the first branch of the culms across various Moso bamboo populations. Each column represents a distinct Moso bamboo population, denoted by codes like AJ, CS, CY, etc., representing 16 sampling regions in China.

	AJ	CS	CY	DA	HS	JZ	LY	RH	TJ	WYS	XA	XN	YA	YF	ZT	YX
**Number of values**	30	29	30	29	30	30	30	30	30	30	30	30	30	30	30	31
**Minimum**	17.0	21.0	18.0	21.0	19.0	18.0	19.0	20.0	18.0	22.0	17.0	21.0	25.0	19.0	17.0	19.0
**Maximum**	25.0	29.0	28.0	30.0	27.0	28.0	28.0	31.0	30.0	32.0	28.0	30.0	36.0	30.0	26.0	29.0
**Range**	8.00	8.00	10.0	9.00	8.00	10.0	9.00	11.0	12.0	10.0	11.0	9.00	11.0	11.0	9.00	10.0
**Mean**	20.6	25.2	23.4	25.2	23.5	22.7	23.5	25.7	23.5	26.9	22.9	24.7	30.9	24.5	21.2	22.4
**Std. Deviation**	2.13	2.14	2.93	2.32	2.21	2.64	2.69	3.06	2.89	2.58	2.71	2.77	2.82	2.69	2.25	2.01
**Coefficient of variation (%)**	10.3	8.51	12.5	9.21	9.41	11.6	11.4	11.9	12.3	9.61	11.8	11.2	9.10	11.0	10.6	8.98

Table S6. Statistical measures of the internode lengths across various Moso bamboo populations. Each column represents a distinct Moso bamboo population, denoted by codes like AJ, CS, CY, etc., representing 16 sampling regions in China.

	AJ	CS	CY	DA	HS	JZ	LY	RH	TJ	WYS	XA	XN	YA	YF	ZT	YX
**Number of values**	619	734	702	752	679	683	704	771	705	806	685	742	928	735	635	668
**Minimum (cm)**	4.40	7.70	6.60	6.00	8.40	6.60	2.90	5.40	0.80	7.20	6.00	7.20	5.50	7.20	7.90	5.57
**Maximum (cm)**	40.4	43.5	37.6	41.5	43.2	44.0	39.2	43.7	43.3	40.0	35.3	46.4	41.0	41.4	42.5	38.4
**Range**	36.0	35.8	31.0	35.5	34.8	37.4	36.3	38.3	42.5	32.8	29.3	39.2	35.5	34.2	34.6	32.8
**Mean (cm)**	23.0	27.8	21.4	24.9	27.0	25.7	21.5	24.5	23.4	25.5	22.7	26.9	24.6	25.7	27.0	23.1
**Std. Deviation**	7.69	8.69	6.27	7.10	8.45	8.23	7.71	8.59	7.63	7.22	6.79	8.43	8.58	7.38	8.46	7.35
**Coefficient of variation (%)**	33.4	31.2	29.3	28.5	31.4	32.0	35.8	35.1	32.7	28.4	29.9	31.4	34.9	28.7	31.3	31.8

Table S7. Statistical measures of the internode diameters across various Moso bamboo populations. Each column represents a distinct Moso bamboo population, denoted by codes like AJ, CS, CY, etc., representing 16 sampling regions in China.

	AJ	CS	CY	DA	HS	JZ	LY	RH	TJ	WYS	XA	XN	YA	YF	ZT	YX
**Number of values**	619	734	702	752	679	683	704	771	706	806	685	742	928	735	635	669
**Minimum (cm)**	6.11	7.85	6.20	6.35	6.10	6.70	6.50	5.90	6.53	6.55	6.20	6.00	5.10	6.00	6.00	6.15
**Maximum (cm)**	11.9	17.2	12.7	14.7	13.8	14.5	16.2	13.4	13.4	14.7	11.8	13.8	14.0	13.7	11.4	12.2
**Range**	5.79	9.32	6.45	8.35	7.70	7.80	9.70	7.50	6.87	8.15	5.60	7.80	8.90	7.70	5.40	6.05
**Mean (cm)**	8.98	11.1	9.14	9.92	10.1	10.4	9.84	9.40	9.65	10.5	8.86	9.82	9.74	10.1	8.53	9.35
**Std. Deviation**	1.09	1.45	1.28	1.58	1.55	1.50	1.55	1.48	1.44	1.65	1.20	1.49	1.68	1.52	0.97	1.19
**Coefficient of variation (%)**	12.1	13.1	14.0	15.9	15.4	14.4	15.7	15.7	14.9	15.7	13.5	15.1	17.3	15.0	11.4	12.8

Table S8. Statistical measures of the internode wall thickness across various Moso bamboo populations. Each column represents a distinct Moso bamboo population, denoted by codes like AJ, CS, CY, etc., representing 16 sampling regions in China.

	AJ	CS	CY	DA	HS	JZ	LY	RH	TJ	WYS	XA	XN	YA	YF	ZT	YX
**Number of values**	619	734	702	752	679	683	704	771	705	806	685	742	928	735	635	668
**Minimum (mm)**	5.50	6.70	5.90	5.80	5.90	3.20	6.20	5.60	5.20	6.30	5.90	5.40	4.30	6.00	5.70	3.20
**Maximum (mm)**	15.8	22.2	19.6	19.3	18.2	20.6	22.2	33.3	21.2	22.0	17.6	18.1	20.0	19.0	16.8	19.5
**Range**	10.3	15.5	13.7	13.5	12.3	17.4	16.0	27.7	16.0	15.7	11.7	12.7	15.7	13.0	11.1	16.3
**Mean (mm)**	9.11	11.2	10.2	10.0	10.0	10.3	10.4	10.2	9.91	11.0	9.47	9.34	9.98	9.79	9.90	9.41
**Std. Deviation**	2.06	2.87	2.49	2.74	2.51	2.67	2.75	3.70	2.58	2.87	2.21	2.33	2.91	2.44	2.44	2.29
**Coefficient of variation (%)**	22.6	25.6	24.4	27.2	25.0	25.9	26.4	36.4	26.0	26.2	23.3	25.0	29.2	24.9	24.6	24.3

Table S9. The mean values of different culm parameters of Moso bamboo used for the correlation analysis. Each column represents a distinct parameter collected from 16 Moso bamboo populations in China.

	D (cm)	*T (mm)*	*D-Slope*	*T-Slope*	*D&T Slope*	*D&L Slope*	*T&L Slope*	*L (cm)*	*H (m)*	*HUFB (m)*	*INUFB*	*IV (cm^3)*	*CVHUFB (cm^3)*	*Ratio-FBRS*	*V_{max} IS*	*V_{max} In-ratio*	*V_{max} H-ratio*
AJ	8.98	0.91	-0.17	0.12	0.44	-0.85	-0.39	23	9.67	4.91	20.6	11212	23946	0.51	12	0.58	0.45
CS	11.1	1.12	-0.19	0.10	0.22	-0.46	-0.21	27.8	17.5	7.33	25.2	2061	54340	0.43	11.8	0.47	0.34
CY	9.14	1.02	-0.16	0.08	0.22	-0.48	-0.21	21.4	13.3	5.19	23.4	1182	28690	0.39	9.8	0.42	0.31
DA	9.92	1.00	-0.18	0.11	0.23	-0.35	-0.15	24.9	15.1	6.39	25.2	1473	37802	0.42	9.5	0.38	0.28
HS	10.1	1.00	-0.19	0.10	0.23	-0.44	-0.21	27.0	14.8	6.53	23.5	1628	39368	0.44	10.6	0.45	0.35
JZ	10.4	1.03	-0.19	0.11	0.28	-0.35	-0.15	25.7	15.2	5.99	22.7	1643	38300	0.39	11.7	0.52	0.39
LY	9.84	1.04	-0.18	0.13	0.41	-0.34	-0.10	21.5	13	5.12	23.5	1309	31166	0.39	13.7	0.58	0.46
RH	9.4	1.02	-0.17	0.11	0.25	-0.34	-0.30	24.5	15	6.65	25.7	1395	37869	0.53	13.3	0.52	0.36
TJ	9.65	0.99	-0.18	0.12	0.31	-0.54	-0.20	23.4	13.7	6.11	23.5	1333	34810	0.45	10.9	0.46	0.32
WYS	10.5	1.10	-0.19	0.08	0.16	-0.27	-0.14	25.5	16.1	7.02	26.9	1753	48252	0.44	11.0	0.41	0.29
XA	8.86	0.95	-0.17	0.10	0.41	-0.52	-0.26	22.7	12.9	5.31	22.9	1132	26484	0.41	11.4	0.50	0.35
XN	9.82	0.93	-0.17	0.10	0.34	-0.45	-0.25	26.9	15.3	6.78	24.7	1476	37195	0.44	12.1	0.49	0.36
YA	9.74	0.90	-0.18	0.10	0.27	-0.47	-0.20	24.6	16.1	7.67	30.9	1425	44423	0.48	14.9	0.48	0.35
YF	10.1	0.98	-0.18	0.10	0.27	-0.35	-0.21	25.7	14.6	6.41	24.5	1519	37874	0.44	10.8	0.44	0.33
ZT	8.53	0.99	-0.13	0.08	0.37	-0.7	-0.23	27.0	14	5.82	21.2	1349	29074	0.42	11.6	0.55	0.44
YX	9.35	0.94	-0.17	0.11	0.34	-0.45	-0.30	23.1	13.8	5.44	22.4	1212	28545	0.40	11.8	0.53	0.40
